# Landscape Genetic Structure of a Streamside Tree Species *Euptelea pleiospermum* (Eupteleaceae): Contrasting Roles of River Valley and Mountain Ridge

**DOI:** 10.1371/journal.pone.0066928

**Published:** 2013-06-25

**Authors:** Xinzeng Wei, Hongjie Meng, Mingxi Jiang

**Affiliations:** 1 Key Laboratory of Aquatic Botany and Watershed Ecology, Wuhan Botanical Garden, Chinese Academy of Sciences, Wuhan, Hubei, People’s Republic of China; 2 University of Chinese Academy of Sciences, Beijing, People’s Republic of China; Instituto de Higiene e Medicina Tropical, Portugal

## Abstract

We used landscape genetics and statistical models to test how landscape features influence connectivity or create barriers to dispersal for a mountain riparian tree species, *Euptelea pleiospermum*. Young leaves from 1078 individuals belonging to 36 populations at elevations of 900–2000 m along upper reaches of four rivers were genotyped using eight nuclear microsatellite markers. We found no evidence for the unidirectional dispersal hypothesis in *E. pleiospermum* within each river. The linear dispersal pattern along each river valley is mostly consistent with the “classical metapopulaton” model. Mountain ridges separating rivers were genetic barriers for this wind-pollinated tree species with anemochorous seeds, whereas river valleys provided important corridors for dispersal. Gene flow among populations along elevational gradients within each river prevails over gene flow among populations at similar elevations but from different rivers. This pattern of gene flow is likely to promote elevational range shifts of plant populations and to hinder local adaptation along elevational gradients. This study provides a paradigm to determine which of the two strategies (migration or adaptation) will be adopted by mountain riparian plants under climate warming.

## Introduction

**Figure 1:**
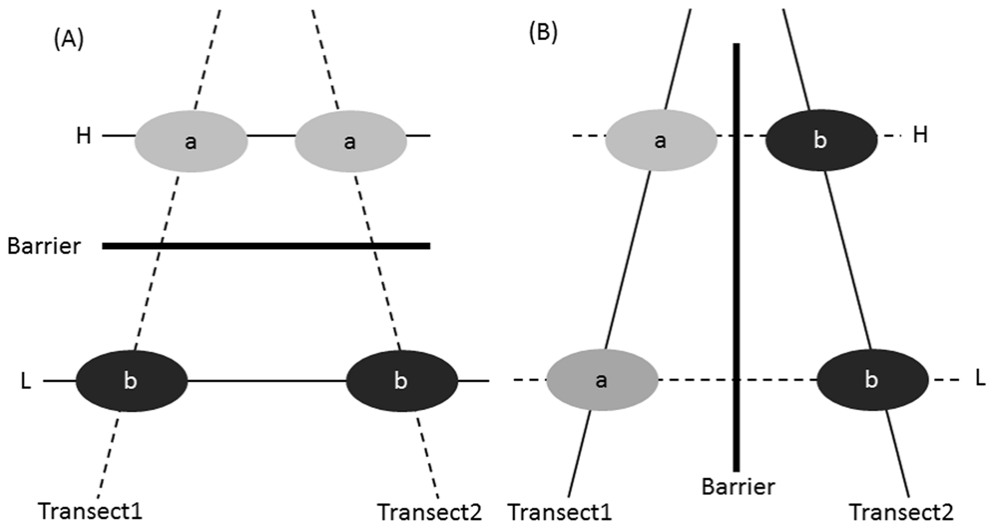
Alternative models of gene flow for mountain plants inhabiting linear habitats along elevation gradients. H, high elevation; L, low elevation. Different lower-case letters in different color ellipses represent different genetic groups.

Migration and adaptation are two main responses of mountain plants to climate warming [1]. The predominant direction of gene flow (along elevational gradients or among similar elevations) is of importance in determining which of the two strategies will be adopted [2], [3], [4], [5]. Here, we propose two models of gene flow (Fig. 1) for mountain plants inhabiting restricted linear habitats (e.g. ridge, streamside, valley or adjacent mountains etc.) that transverse elevation gradients. First, gene flow among populations at similar elevations but along adjacent linear habitats is higher than that within the same linear habitat at different elevations (Fig. 1A) because of similar microclimate and flowering times (phenology effect). Support for this model of gene flow has been shown for *Poa hiemata* [2] and *Castanopsis eyrei* [3]. Second, gene flow is more likely to occur within each linear habitat than between them (Fig. 1B) because of barriers (e.g. valley or ridge) between linear habitats along elevational gradients (geographic barrier effect). Support for this model has been found in *Ainsliaea faurieana* [6] and *Silene ciliata* [5]. The first model of gene flow (Fig. 1A) could promote local adaptation within an elevational zone but also potentially hinder elevational range shifts of plant populations, whereas the second model (Fig. 1B) would render the opposite trend.

For mountain riparian plants, gene flow is influenced by mountain landscape characteristics, such as elevation, stream flow, river valley and mountain ridge [6], [7], [8]. As dispersal is an important determinant of the level and direction of gene flow, it is essential for understanding how landscape features influence connectivity or create barriers to dispersal [9], [10], [11].

River valleys are long-recognized corridors for riparian or aquatic plants, which are characterized by linear distribution patterns [12], [13]. Previous studies have proposed six hypothetical models about the patterns of dispersal and connectivity between linearly arranged populations [12], [14]. The two extreme models are “spatially extended population” and “regional ensemble” [15] with free and without any ongoing gene flow among populations, respectively. For species characterized predominantly by unidirectional water-mediated dispersal, the linear asymmetrical adjacent flow model and the linear asymmetrical non-adjacent flow model were proposed [16]. The two models predict that downstream populations may harbor higher genetic diversity [17], and a few studies on riparian plants have confirmed this prediction [6], [18], [19]. However, wind- or animal-mediated upstream seed and/or pollen dispersal, which are likely to be promoted by valleys (corridors) along rivers, may weaken or counteract this cumulative effect [8], [12], [20], [21], [22]. Therefore, the two bidirectional dispersal models, the “classical stepping-stone” model and the “classical metapopulation” model, seem to be more common in studied linear populations, especially the latter.

Mountain ridge is the predominant factor causing limited gene flow and genetic differentiation in mountain plants [6], [23]. As streamside in mountainous areas is always at the bottom of a deep valley, linear distribution patterns constrained by highlands on both sides render populations with sharp boundaries and strong natural fragmentation; such spatial structure hampers gene flow among rivers. Therefore, populations at similar elevation along different rivers may be genetically separated by ridges. The linear distribution pattern can, in turn, facilitate gene flow within river valleys, especially for wind-dispersed plants. Therefore, we expected that gene flow of mountain riparian plants would be consistent with the second model (Fig. 1B).

*Euptelea pleiospermum* Hook. f. et Thoms (Eupteleaceae) is a diploid (2*n* = 28) [24], deciduous, broad-leaved Tertiary-relict tree species endemic to China, India and Burma [25]. In China, this species distributes across wide altitudinal ranges along streamsides (720–3600 m a.s.l.) [25]. As other riparian plant species, *E. pleiospermum* is restricted to linear suitable habitats. It is wind-pollinated and flowers in early spring before leaf-out. Adult trees produce numerous indehiscent fruits. The small and light samaras are dispersed first by gravity and secondarily by wind and/or water [26]. For this species, Li [27] has observed water-mediated seed dispersal in a downstream direction by simulation experiments with dyed real and artificial fruits.

The purpose of the present study is to examine the landscape genetic structure of *Euptelea pleiospermum* populations located along four main rivers (elevation gradients) within one mountain. Specially, the following questions were addressed: (1) does the recognized water-mediated seed dispersal result in genetic diversity accumulation in downstream populations? If not, which of the other linear models is suitable for populations within rivers? (2) Do river valleys and mountain ridges act as migration corridors and genetic barriers, respectively?

## Materials and Methods

### Ethics Statement

All necessary permits were obtained for the described field studies. Mr. Jingyuan Yang from the Administration Bureau of the Shennongjia National Nature Reserve issued the permission for each location.

### Study Area and Sample Collection

**Figure 2:**
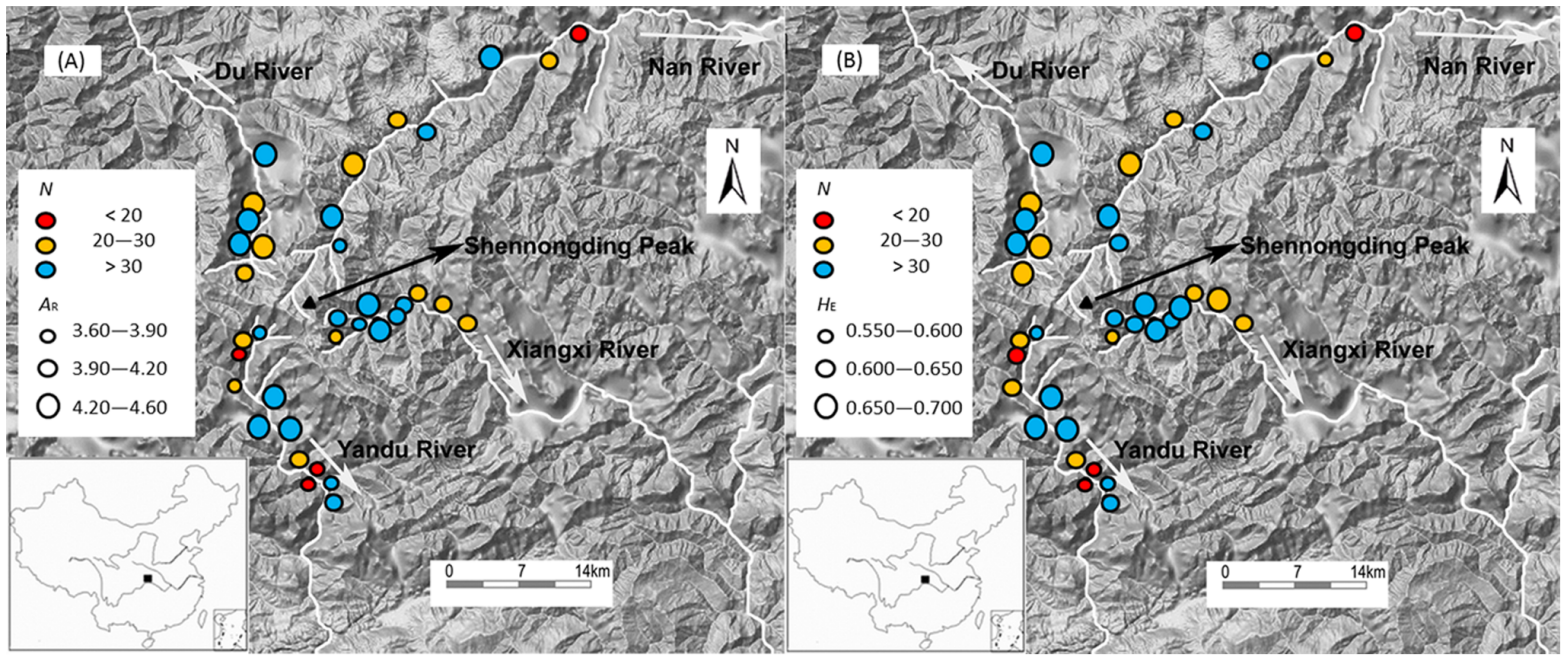
Distribution of genetic diversity (A, allelic richness (*A*_R_) and B, expected heterozygosity (*H*_E_)) in 36 *Euptelea pleiospermum* populations along the four main rivers in the Shennongjia Mountains. Circles indicate sampling locations, colors represent sample size (*N*), and circle size is proportional to the values of *A*_R_ or *H*_E_. The white arrows indicate the flow direction of the four rivers (white lines).

The study area is located in mountain riparian forests of the Shennongjia National Nature Reserve (SNNR) (31°21′20″–31°36′20″ N, 110°03′05″–110°33′50″ E), central China (Fig. 2). Its summit Shennongding (3105.4 m) is the highest peak in central China [28]. SNNR is an important part of the south-central China biodiversity hot-spot and is rich of Tertiary relicts and endemic plants [29], [30]. There are four main river systems in the Shennongjia Mountains: Yandu River and Xiangxi River on the south-facing slope, which are tributaries of the Yangtze River, and Nan River and Du River on the north-facing slope, which are the tributaries of the Han River (Fig. 2). *Euptelea pleiospermum* is a common tree species in riparian forests at elevations between ∼1200 and ∼1900 m in this area [31].

Fieldwork was carried out in April and May 2008. Young leaves were collected and immediately dried in a 10 cm ×5 cm plastic bag containing silica gel. This was repeated every ∼100 m from high to low altitude along the four rivers. The sampling range depended on the altitudinal range of *E. pleiospermum* along each river. In this study, a total of 36 populations were sampled with six to 12 populations for each river (Fig. 2). Because of the sporadic distribution at some altitudes, sample sizes were sometimes relatively low, ranging from six to 61 per population (Table 1 and Fig. 2). A total of 1078 individuals were analyzed in this study.

**Table 1:** Characteristics of the 36 populations of*Euptelea pleiospermum* sampled, including river, population name (Pop.), altitude, latitude (Lat.), longitude (Long.), sample size (*N*), mean number alleles per locus (*A*), allelic richness (*A*_R_), expected and observed heterozygosity (*H*_E_ and *H*_O_), and inbreeding coefficient (*F*_IS_).

River	Pop.	Altitude (m)	Lat. (N)	Long. (E)	*N*	*A*	*A*_R_	*H*_E_	*H*_O_	*F*_IS_
Yandu	Y1	2013	31°19.210′	110°26.269′	33	6.00	3.65	0.558	0.386	**0.267**
	Y2	1962	31°19.228′	110°26.236′	24	6.00	4.09	0.635	0.568	0.099
	Y3	1805	31°19.331′	110°26.048′	6	3.88	3.88	0.606	0.521	**0.245**
	Y4	1710	31°19.550′	110°26.031′	28	5.25	3.76	0.621	0.536	**0.145**
	Y5	1640	31°19.569′	110°25.857′	40	6.63	4.38	0.686	0.581	**0.148**
	Y6	1575	31°19.774′	110°25.930′	61	6.75	4.33	0.657	0.518	**0.165**
	Y7	1471	31°19.960′	110°25.512′	41	6.63	4.45	0.687	0.534	**0.226**
	Y8	1320	31°20.106′	110°25.103′	28	6.00	4.09	0.643	0.522	**0.173**
	Y9	1270	31°20.404′	110°24.810′	11	4.63	3.80	0.558	0.455	**0.169**
	Y10	1101	31°19.541′	110°23.851′	10	4.25	3.68	0.575	0.425	**0.281**
	Y11	1005	31°19.516′	110°23.750′	37	6.25	3.79	0.555	0.463	**0.169**
	Y12	980	31°19.351′	110°23.615′	32	6.13	4.18	0.613	0.430	**0.268**
Xiangxi	X1	2003	31°24.286′	110°19.525′	22	5.00	3.64	0.577	0.631	–0.104
	X2	1910	31°24.387′	110°19.715′	42	5.88	3.98	0.639	0.512	**0.183**
	X3	1850	31°25.095′	110°19.914′	39	6.13	3.74	0.636	0.535	**0.148**
	X4	1715	31°24.948′	110°20.266′	32	6.63	4.41	0.664	0.586	**0.096**
	X5	1690	31°25.057′	110°20.338′	31	6.50	4.58	0.659	0.411	**0.335**
	X6	1520	31°25.438′	110°21.124′	33	6.25	4.18	0.611	0.564	**0.072**
	X7	1478	31°25.629′	110°21.487′	47	6.50	4.12	0.658	0.545	**0.135**
	X8	1389	31°26.015′	110°21.534′	25	5.13	4.01	0.646	0.540	**0.145**
	X9	1270	31°25.886′	110°22.754′	22	5.50	3.96	0.651	0.483	**0.271**
	X10	1190	31°26.586′	110°23.372′	29	6.13	4.15	0.645	0.509	**0.217**
Nan	N1	1904	31°29.936′	110°19.291′	36	5.88	3.71	0.622	0.604	**0.027**
	N2	1875	31°30.182′	110°19.523′	31	6.13	4.28	0.671	0.589	**0.126**
	N3	1725	31°32.327′	110°20.158′	25	5.75	4.27	0.656	0.530	**0.165**
	N4	1675	31°33.736′	110°21.564′	30	5.88	4.03	0.613	0.504	**0.137**
	N5	1521	31°36.260′	110°24.575′	37	6.63	4.13	0.611	0.520	**0.133**
	N6	1410	31°36.899′	110°25.569′	32	6.88	4.24	0.635	0.508	**0.178**
	N7	1360	31°37.079′	110°26.047′	24	5.75	3.93	0.590	0.521	**0.124**
	N8	1290	31°37.675′	110°27.039′	15	5.25	4.08	0.629	0.558	**0.153**
Du	D1	2070	31°28.307′	110°09.118′	26	5.88	4.14	0.654	0.572	**0.120**
	D2	1940	31°28.353′	110°08.966′	28	6.00	4.45	0.665	0.549	**0.145**
	D3	1860	31°28.134′	110°08.860′	37	6.38	4.36	0.666	0.588	**0.104**
	D4	1769	31°28.060′	110°08.596′	32	6.25	4.22	0.667	0.633	**0.052**
	D5	1682	31°27.721′	110°08.117′	20	6.00	4.41	0.656	0.619	**0.049**
	D6	1468	31°28.473′	110°01.316′	32	6.38	4.27	0.679	0.58	**0.117**

Bold values indicate significant departure from Hardy-Weinberg equilibrium.

### DNA Extraction and Microsatellite Genotyping

All samples were stored at 4°C pending DNA extraction. Total DNA was extracted from approximately 0.5 g dried leaves using a modified CTAB method [32]. The DNA sample was diluted to 5–10 ng µL^−1^. Fourteen published primer pairs [33] were tested using 36 individuals (one individual randomly chosen from each population) and eight nuclear microsatellite loci (EP021, EP036, EP059, EP081, EP087, EP091, EP278, and EP294) that detected a suitable level of polymorphism were selected to determine genotypes of the 1078 samples.

The microsatellite genotyping procedure was performed as described by Wei and Jiang [34]. PCR amplifications were carried out in a total volume of 10 µL consisting of 10–50 ng of template DNA, 1 µL 10× reaction buffers, 1.5 mM of MgCl_2_, 0.2 mM of dNTPs, 0.25 µM of each primer, and 0.5 U of *Taq* polymerase (Tiangen Biotech Co., LTD., Beijing, China ). PCR reaction was conducted in a PTC-100 thermal cycler (MJ Research, Inc., Cambridge, MA, USA). PCR reactions were performed with the following cycle profile: an initial denaturation at 94°C for 5 min; then 35 cycles of denaturation at 94°C for 50 s, annealing at 56°C for 50 s and extension at 72°C for 1 min; and final extension at 72°C for 10 min. After amplification, identification of the alleles was based on the position of the fragments in relation to a 25-bp marker ladder on a polyacrylamide gel of high resolution with silver stain.

### Data Analysis

GENETIX 4.05 [35] was used to calculate the following population diversity indices: mean number of alleles per locus (*A*), expected (*H*_E_) and observed (*H*_O_) heterozygosity, and inbreeding coefficient (*F*_IS_) across all loci and locus-population combinations. FSTAT 2.9.3.2 [36] was used to estimate allelic richness (*A*_R_) for each population and to test for linkage disequilibrium (LD). Departure from Hardy-Weinberg equilibrium (HWE) was tested by Fisher’s exact test in GENEPOP version 4.0 [37]. The significant values for the LD were corrected for multiple comparisons by Bonferroni correction [38]. We used the program MICRO-CHECKER [39] to test for null alleles.

To reveal the pattern of genetic diversity along rivers, regression analyses between genetic diversity parameters (*H*_E_ and *A*_R_) and the position of populations along the course of the river were conducted using the SPSS statistics program (SPSS 15.0 for Windows; SPSS Inc., Chicago, IL, USA).

To provide direct evidence for the dispersal pattern along each river, contemporary and historical migration rates between populations along each river were calculated by using the programs BayesAss version 1.3 [40] and MIGRATE version 3.2.2.windows [41], respectively. BayesAss was run using a Markov chain Monte Carlo (MCMC) length of 3,000,000 with a burn-in period of 1,000,000 (initial conditions of Δ*p*, Δ*m*, and Δ*F* = 0.15). MIGRATE uses Bayesian inference with long chains (500,000 steps sampled, 5,000 steps recorded) and 1,000 burn-in per chain. We then compared mean values of downstream and upstream migration rates along each river valley, and significance was tested with a paired-samples *t*-test.

Pairwise population differentiation was estimated at two scales: among populations and among rivers. *F*_ST_ [42] and *D*_EST_ [43] were calculated using FSTAT 2.9.3.2 [36] and SMOGD 1.2.5 [44], respectively. Significance tests for *F*_ST_ were based on 1000 randomizations. We compare genetic differentiation among populations from different rivers and that among populations within the same river (inter-*F*_ST_ vs intra-*F*_ST_ and inter-*D*_EST_ vs intra-*D*_EST_, respectively), and significance was tested by performing an independent-samples *t*-test.

To analyze the level and partitioning of genetic variation among geographical groups, among populations within geographical groups, and within populations, analysis of molecular variance (AMOVA) [45] was performed with 1000 permutations in ARLEQUIN version 3.1 [46]. There were two kinds of geographical groups: populations along each river (Yandu, Xiangxi, Nan, and Du) and populations in each altitudinal zone (12, from 900 m to 2000 m).

To test whether the individuals from the same river valley or from similar altitudes can be clustered into genetically homogeneous groups, nonspatial and spatially sensitive Bayesian clustering were implemented by using STRUCTURE version 2.3.3 [47], [48], [49], [50] and TESS version 2.3.1 [51]. Previous STRUCTURE analysis employed a Markov chain Monte Carlo (MCMC) approach to cluster individuals into *K* panmictic groups without *a priori* assignment of individuals to sampling locations and by minimizing deviations from Hardy-Weinberg equilibrium and linkage equilibrium. The LOCPRIOR model, recently developed by Hubisz et al. [52], allowed us to infer weak population structure with the assistance of sample group information. To guide an empirical estimate of the number of genetic populations, we performed five replicates of each simulation from *K* = 1 to *K* = *N*_P_ +3 (*N*_P_, the number of sampling locations), with a burn-in period of 100,000, and MCMC iterations of 1,000,000, based on the LOCPRIOR model and the admixture model with correlated allele frequencies. We applied Evanno et al.’s [53] *ad hoc* statistic, *ΔK*, to calculate the uppermost hierarchical level of structure (*K*). For the spatially sensitive TESS analysis (admixture model: BYM; spatial interaction parameter: 0.6), we set 10 runs per *K*_max_ (maximal number of clusters), for *K*_max_ ranging from two to six, with 300,000 total sweeps and a burn-in of 100,000.We used the program DISTRUCT version 1.1 [54] to create bar charts for the output data derived from STRUCTURE and TESS analyses.

To test for isolation by distance (IBD) [55], the correlation between genetic distances (*F*_ST_/(1–*F*_ST_)) and altitudinal distances (logarithmically transformed) among populations was executed using the Mantel test implemented in GenAlEx 6.41 [56]. We also performed partial Mantel test to check for the effect of altitudinal distance after accounting for geographical distance by using FSTAT 2.9.3.2 [36]. The IBD tests were performed at two spatial scales: each of the four rivers and the whole Shennongjia Mountains. Latitude and longitude coordinates for each population were used to calculate pairwise geographical distances between populations.

## Results

### Microsatellite Polymorphism, Hardy-Weinberg Equilibrium and Linkage Disequilibrium

A total of 102 alleles at eight nuclear microsatellite loci were revealed across 1078 individuals of 36 sampled populations of *E. pleiospermum*. At the species level, all eight nuclear microsatellite markers were highly polymorphic, having 6–29 alleles per locus with an average of 12.8. At the intra-population level, mean number of alleles per locus (*A*) ranged from 3.88 to 6.88, and allelic richness (*A*_R_) varied from 3.64 to 4.58 (Table 1 and Fig. 2A). The expected heterozygosity (*H*_E_) ranged from 0.555 to 0.687 (Fig. 2B), and observed heterozygosity (*H*_O_) ranged from 0.386 to 0.633 (Table 1). The level of inbreeding for each population ranged from –0.104 to 0.335 and significant inbreeding coefficients (*F*_IS_) were detected in 34 of 36 populations (Table 1).

The test for HWE found that 135 of 288 locus-population combinations were significant, including 32 at locus EP021, 21 at EP036, three at EP059, 17 at EP081, 22 at EP087, nine at EP091, eight at EP278 and 23 at EP294. However, no locus displayed consistent deviation from HWE across all populations. Furthermore, MICROCHECKER revealed potential null alleles in five of the 36 populations, with three at locus EP021 and two at EP297. This suggested that departure from HWE is potentially resulted from both technical (e.g. null allele) and biological (e.g. inbreeding) reasons. The test for genotypic disequilibrium found that only one (EP036×EP294) of 28 locus pairs showed significant genotypic disequilibrium in 12 populations. Because no consistent genotypic disequilibrium was found between any locus pair across all populations, all loci were used for further analyses.

### Linear Population Model along Rivers

Linear regression analyses did not reveal any significant negative relationship between genetic diversity and the position of populations along the course of the rivers (Table 2 and Fig. 2), suggesting that there was no accumulation of genetic diversity in downstream populations. In accordance with this result, we found no significant difference between downstream and upstream migration rates, although the former was a little higher than the latter along all the four rivers (Table 3, Table S1 and Table S2).

**Table 2:** Correlation between genetic diversity (*A*_R_, Allelic richness and *H*_E_, expected heterozygosity) and the position of populations along the course of the river at different spatial scales.

Position	*A*_R_		*H*_E_	
	*r*	*p*	*r*	*p*
Shennongjia Mountain	0.001	0.995	−0.188	0.272
Yandu River	0.052	0.871	−0.197	0.540
Xiangxi River	0.295	0.408	0.477	0.163
Nan River	0.127	0.764	−0.467	0.244
Du River	0.122	0.818	0.646	0.166

**Table 3:** Comparison of downstream and upstream migration rates of*Euptelea pleiospermum* along each of the four rivers (Mean ± S.E.).

	Contemporary migration rate				Historical migration rate		
	Downstream	Upstream	*p*		Downstream	Upstream	*p*
Yandu River	0.023±0.006	0.016±0.005	0.461		24.05±3.33	17.68±1.51	0.107
Xiangxi River	0.015±0.007	0.010±0.005	0.563		21.35±2.56	20.91±2.59	0.913
Nan River	0.029±0.010	0.015±0.009	0.313		22.80±2.21	18.14±1.82	0.127
Du River	0.038±0.017	0.023±0.006	0.351		25.52±4.80	24.36±4.45	0.868

Contemporary and historical migration rates were calculated by using the programs BayesAss version 1.3 MIGRATE version 3.2.2., respectively.

Moderate levels of pairwise differentiation (*F*_ST_ and *D*_EST_) were found between populations within each river valley. Significance test revealed that most values of *F*_ST_ were significantly different from zero (Table S3).

Mantel test detected significant IBD within the Yandu River and Du River (Table 4). However, after controlling for geographical distance, the partial Mantel test detected no significant relationships between genetic and altitudinal distances within any of the four rivers (Table 4).

**Table 4:** Results of Mantel tests and partial Mantel test of pairwise genetic distance (*F*_ST_/(1–*F*_ST_)) and the natural logarithm of altitudinal distance for *Euptelea pleiospermum* at different spatial scales.

	Mantel test		Partial Mantel test	
Altitudinal distance	*r*	*p*	*r*	*p*
Shennongjia Mountain	0.149	0.000	0.142	0.000
Yandu River	0.517	0.000	0.175	0.163
Xiangxi River	0.118	0.439	0.090	0.560
Nan River	0.271	0.163	−0.029	0.886
Du River	0.596	0.019	0.389	0.169

### Roles of Mountain Ridges and River Valley

When individuals from the same river were seen as one population, all pairwise *F*_ST_ values between rivers were significantly different from zero, and the pairwise *D*_EST_ values also showed similar levels of genetic differentiation (Table 5).

**Table 5:** Matrix of pairwise differentiation (*D*_EST_, above diagonal and *F*_ST_, below diagonal) among rivers.

	Yandu River	Xiangxi River	Nan River	Du River
Yandu River	–	0.016	0.018	0.016
Xiangxi River	0.013	–	0.019	0.030
Nan River	0.026	0.015	–	0.027
Du River	0.012	0.015	0.026	–

All values of *F*_ST_ are significant based on permutation tests following a sequential Bonferroni correction.

We found moderate levels of genetic differentiation among most pair of populations from different rivers (Table S3). Furthermore, average *F*_ST_ values were higher for inter-river population pairs than for intra-river population pairs (*F*_ST_ inter: 0.069±0.001; *F*_ST_ intra: 0.061±0.003, *P* = 0.007), and *D*_EST_ values showed the same trend (*D*_EST_ inter: 0.091±0.002; *D*_EST_ intra: 0.082±0.004, *P* = 0.032) (See Table S3 for details).

The results of the AMOVA were shown at Table 6. There was significant differentiation among different rivers (0.99%; *P*<0.001). However, no significant differentiation was found among populations located at different altitudinal zones (0.20%; *P* = 0.206).

**Table 6:** Effects of mountain ridges (A) and river valleys (B) on differences among 36*Euptelea pleiospermum* populations as determined by analysis of molecular variance (AMOVA).

Source of variation	d.f.	Sum of squares	Variance components	Percentage of variation	*p*
(A)					
Among rivers	3	85.401	0.028	0.99	<0.001
Among populations within river valleys	32	411.263	0.173	6.20	<0.001
Within populations	2120	4598.853	2.594	92.81	<0.001
Total	2155	5995.517	2.795		
(B)					
Among altitudinal zones	11	170.286	0.005	0.20	0.206
Among populations within altitudinal zones	24	326.378	0.189	6.79	<0.001
Within populations	2120	5498.853	2.594	93.02	<0.001
Total	2155	5995.517	2.788		

**Figure 3:**
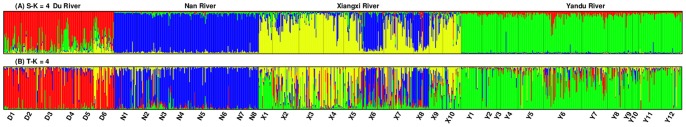
Population structure of*Euptelea pleiospermum* estimated by the programs STRUCTURE 2.3.4 (A) and TESS 2.3.1 (B). Each individual is represented by a thin vertical line, which is partitioned into four colored segments that represent the individual’s estimated membership fractions in the four clusters. Black vertical lines separate individuals of different populations. Labels below the plot provide population codes, which are the same as in Table 1. Labels above the plot (Du River, Nan River, Xiangxi River and Yandu River) are sampling information.

With the four rivers as sample group information, STRUCTURE analysis revealed a clear peak of Evanno et al.’s [53] *ad hoc* statistic *ΔK* at *K* = 4. The bar plot assuming *K* = 4 showed that individuals from different rivers were clustered into distinct groups (Fig. 3A). However, the same analysis did not clearly detect genetic structure when the 12 altitudinal zones were used in the LOCPRIOR model (data not shown). Results from TESS paralleled those obtained from STRUCTURE, although there are some minor differences (Fig. 3).

Both Mantel and partial Mantel tests found significant correlations between *F*_ST_-based genetic distances and altitudinal distances within the whole Shennongjia Mountains (Table 4).

## Discussion

### Linear Population Model within Rivers

We found no evidence for the unidirectional dispersal hypothesis in *E. pleiospermum* (Tables 2 and 3, Fig. 2), although water-mediated seed dispersal in a downstream direction has been observed [27]. In other words, upstream dispersal of pollen and seed mediated by wind are efficient mechanisms to eliminate the downstream accumulation of genetic diversity caused by water-mediated seed dispersal. Besides upstream dispersal, Honnay et al. [22] recently pointed out that higher seed recruitment opportunities in upstream habitats due to density dependence of recruitment is another explanation. However, this seems not the case for *E. pleiospermum* in the study area, because the pattern of genetic diversity along the Yandu River supports the central-marginal hypothesis [57].

The “regional ensemble” and “classical stepping-stone” models were rejected because IBD was not observed within any of the four rivers (Table 4), indicating that gene flow along each river was across a wide elevational range. The “spatially extended population”, a single genetically uniform panmictic unit, was also rejected because most of the genetic differentiation values among populations were significant. As inferred above, the bidirectional long-distance dispersal within each river valley and the significant genetic differentiation among populations indicated that the linear dispersal pattern for *E. pleiospermum* is most consistent with the “classical metapopulaton” model. This model was also confirmed in another riparian tree species, *Populus nigra* [20]; anemochory is the most important dispersal mode for this species as well.

### Contrasting Roles of Mountain Ridges and River Valleys

Our results suggested that mountain ridges and river valleys played contrasting roles in shaping the genetic structure of *E. pleiospermum* at the landscape scale. Mountain ridges between rivers can be recognized as genetic barriers, while river valleys act as corridors for gene flow. In other words, gene flow of *E. pleiospermum* is consistent with the second model (Fig. 1B).

Four results lead to the conclusion that mountain ridges act as genetic barriers. First, individuals from each river were clustered into distinct groups (Fig. 3). Furthermore, the 12 altitudinal zones were not clustered into different groups by STRUCTURE analysis and this result suggested that populations from different rivers at similar elevations could not be clustered into the same group. Second, the *F*_ST_ and *D*_EST_ values for inter-river comparisons were higher than intra-river comparisons. This has been found in another mountain riparian plant, *Ainsliaea faurieana* [6]. Third, we found significant IBD in the whole Shennongjia Mountains but lack of IBD within all four rivers (Table 4). Fourth, when each river was seen as a population, we detected significant genetic differentiation among them (Table 5). This was confirmed by the results of AMOVA, which showed that there was significant differentiation among rivers, whereas no significant differentiation was found among altitudinal zones (Table 6). Mountain ridges have been found as genetic barriers for another wind dispersed tree species, *Betula maximowicziana* [23].

However, unambiguous evidence for gene flow among rivers is that the X6 (1520 m) and X8 (1389 m) populations from Xiangxi River were clustered into the Nan River group, as revealed by STRUCTURE and TESS analyses (Fig. 3). This can be partly explained by the *ex situ* conservation performed with plant materials collected from the Nan River. There is one national highway connecting the two rivers, and there was an *ex situ* conservation site at ∼1400 m along the Xiangxi River in the past. Furthermore, it has been reported that roads can serve as dispersal corridors for plants [58], [59]. However, it is not yet clear why X1 (2003 m) and X9 (1270 m) populations were clustered into the Yandu River group (Fig. 3B).

Our results also support that linear riparian zones in deep valleys provide important corridors for gene flow. First, as mentioned above, lack of IBD in each river indicated that gene flow along the river valley was not restricted (Table 4). This has previously been reported for riparian and aquatic plants [6], [14]. Second, as mentation above, the *F*_ST_ and *D*_EST_ values for inter-river comparisons were higher as compared to intra-river comparisons. Third, the Bayesian clustering approach divided individuals from the four rivers into four distinct groups (Fig. 3), indirectly indicating that gene flow within each river was relatively high.

### Conclusions and Implications

We found no accumulation of genetic diversity at the downstream populations of *E. pleiospermum* within each of the four rivers. This was supported by the estimates of contemporary and historical migration rates, which revealed that the downstream migration rate was not significantly higher than that in upstream direction. The linear dispersal pattern along each river valley was mostly consistent with the “classical metapopulaton” model. Mountain ridges among rivers were genetic barriers for this wind-pollinated tree species with anemochorous seeds, whereas river valleys provided important corridors for dispersal. In summary, the streamside population of *E. pleiospermum* is consistent with the second model of gene flow (Fig. 1B) for mountain plants restricted to linear habitats along elevational gradients. Here, it should be mentioned that the variation of sample size, especially small number of sample in some of the populations (less than 20), may influence in our conclusions.

Gene flow among *E. pleiospermum* populations along elevational gradients within each river prevailed over gene flow among populations at similar elevations but from different rivers. This pattern of gene flow could promote elevational range shifts of plant populations but potentially hinder local adaptation along elevational gradients. Therefore, range shift along elevations is the most likely strategy for *E. pleiospermum* in the face of climate warming. As mountain riparian zones are crucial refuges for tertiary-relict plants [60], [61], [62], [63], this study provides a paradigm to determine which of the two strategies (migration or adaptation) will be adopted by these plants under climate warming.

## Supporting Information

Table S1 **Contemporary migration rates between** ***Euptelea pleiospermum*** **populations along each river.**
(DOC)

Table S2 **Historical migration rates between** ***Euptelea pleiospermum*** **populations along each river.**
(DOC)

Table S3 **Pair-wise population** ***D*** **_EST_ (above the diagonal) and** ***F*** **_ST_ (below the diagonal).**
(XLS)
