# Supplementary material for: Landscape Genetic Structure of a Streamside Tree Species Euptelea pleiospermum (Eupteleaceae): Contrasting Roles of River Valley and Mountain Ridge
Source: PLoS One. 2013 Jun 25;8(6):e66928. doi: 10.1371/journal.pone.0066928 (PMC3692547; doi:10.1371/journal.pone.0066928)
Supplement: Table S1 — Contemporary migration rates between Euptelea pleiospermum populations along each river. (DOC) [file pone.0066928.s001.doc]

**Table S1** Contemporary migration rates between *Euptelea pleiospermum* populations along each river obtained using the program BayesAss version 1.3 (Wilson & Rannala 2003) from initial conditions of Δ*p*, Δ*m*, and Δ*F* = 0.15. Means of the posterior distribution for, m, the migration rate per generation, into each population are shown for each population pair. Migration rates are estimated as the proportion of individuals in row populations that are derived from populations in columns (Above diagonal, up-to-down migration rate; below diagonal, down-to-up migration rate). Values along the diagonal are representive of the proportion of individuals within a population derived from that population.

|  | Y2000 | Y1900 | Y1800 | Y1700 | Y1600 | Y1500 | Y1400 | Y1300 | Y1200 | Y1100 | Y1000 | Y900 |
| --- | --- | --- | --- | --- | --- | --- | --- | --- | --- | --- | --- | --- |
| Y2000 | 0.952 | 0.022 | 0.026 | 0.002 | 0.010 | 0.001 | 0.007 | 0.006 | 0.010 | 0.011 | 0.001 | 0.014 |
| Y1900 | 0.002 | 0.683 | 0.015 | 0.001 | 0.003 | 0.001 | 0.003 | 0.005 | 0.010 | 0.011 | 0.001 | 0.003 |
| Y1800 | 0.002 | 0.008 | 0.711 | 0.001 | 0.005 | 0.001 | 0.048 | 0.236 | 0.113 | 0.012 | 0.001 | 0.004 |
| Y1700 | 0.024 | 0.233 | 0.108 | 0.986 | 0.078 | 0.002 | 0.008 | 0.005 | 0.018 | 0.013 | 0.001 | 0.006 |
| Y1600 | 0.002 | 0.009 | 0.016 | 0.001 | 0.677 | 0.001 | 0.004 | 0.004 | 0.011 | 0.012 | 0.001 | 0.004 |
| Y1500 | 0.005 | 0.012 | 0.031 | 0.001 | 0.196 | 0.989 | 0.218 | 0.031 | 0.090 | 0.026 | 0.002 | 0.007 |
| Y1400 | 0.002 | 0.006 | 0.016 | 0.001 | 0.004 | 0.001 | 0.685 | 0.004 | 0.011 | 0.012 | 0.001 | 0.003 |
| Y1300 | 0.002 | 0.006 | 0.015 | 0.001 | 0.007 | 0.001 | 0.007 | 0.689 | 0.012 | 0.032 | 0.001 | 0.227 |
| Y1200 | 0.002 | 0.005 | 0.015 | 0.001 | 0.003 | 0.001 | 0.003 | 0.004 | 0.691 | 0.010 | 0.001 | 0.003 |
| Y1100 | 0.002 | 0.005 | 0.015 | 0.001 | 0.004 | 0.001 | 0.003 | 0.004 | 0.009 | 0.695 | 0.001 | 0.003 |
| Y1000 | 0.003 | 0.005 | 0.016 | 0.001 | 0.010 | 0.001 | 0.012 | 0.008 | 0.014 | 0.154 | 0.988 | 0.046 |
| Y900 | 0.002 | 0.006 | 0.015 | 0.001 | 0.004 | 0.001 | 0.003 | 0.004 | 0.011 | 0.012 | 0.001 | 0.678 |
|  |  |  |  |  |  |  |  |  |  |  |  |  |
|  | X2000 | X1900 | X1800 | X1700 | X1600 | X1500 | X1400 | X1300 | X1200 | X1100 |  |  |
| X2000 | 0.681 | 0.001 | 0.001 | 0.004 | 0.002 | 0.002 | 0.002 | 0.002 | 0.003 | 0.005 |  |  |
| X1900 | 0.016 | 0.983 | 0.001 | 0.009 | 0.003 | 0.003 | 0.013 | 0.002 | 0.006 | 0.211 |  |  |
| X1800 | 0.025 | 0.002 | 0.989 | 0.248 | 0.005 | 0.002 | 0.002 | 0.002 | 0.003 | 0.010 |  |  |
| X1700 | 0.005 | 0.001 | 0.001 | 0.689 | 0.002 | 0.002 | 0.002 | 0.002 | 0.003 | 0.004 |  |  |
| X1600 | 0.007 | 0.003 | 0.001 | 0.011 | 0.978 | 0.004 | 0.005 | 0.002 | 0.003 | 0.006 |  |  |
| X1500 | 0.005 | 0.001 | 0.001 | 0.004 | 0.002 | 0.977 | 0.009 | 0.002 | 0.003 | 0.004 |  |  |
| X1400 | 0.241 | 0.003 | 0.001 | 0.009 | 0.002 | 0.003 | 0.936 | 0.003 | 0.020 | 0.029 |  |  |
| X1300 | 0.005 | 0.001 | 0.001 | 0.016 | 0.002 | 0.002 | 0.020 | 0.984 | 0.003 | 0.004 |  |  |
| X1200 | 0.009 | 0.002 | 0.001 | 0.004 | 0.002 | 0.003 | 0.007 | 0.002 | 0.952 | 0.030 |  |  |
| X1100 | 0.005 | 0.001 | 0.001 | 0.005 | 0.002 | 0.002 | 0.003 | 0.002 | 0.003 | 0.697 |  |  |
|  |  |  |  |  |  |  |  |  |  |  |  |  |
|  | N1900 | N1800 | N1700 | N1600 | N1500 | N1400 | N1300 | N1200 |  |  |  |  |
| N1900 | 0.987 | 0.023 | 0.003 | 0.004 | 0.003 | 0.006 | 0.006 | 0.070 |  |  |  |  |
| N1800 | 0.002 | 0.677 | 0.002 | 0.003 | 0.003 | 0.008 | 0.004 | 0.010 |  |  |  |  |
| N1700 | 0.002 | 0.262 | 0.982 | 0.007 | 0.005 | 0.052 | 0.006 | 0.159 |  |  |  |  |
| N1600 | 0.002 | 0.007 | 0.003 | 0.931 | 0.018 | 0.021 | 0.007 | 0.011 |  |  |  |  |
| N1500 | 0.003 | 0.013 | 0.003 | 0.016 | 0.959 | 0.220 | 0.110 | 0.027 |  |  |  |  |
| N1400 | 0.002 | 0.004 | 0.002 | 0.003 | 0.003 | 0.679 | 0.004 | 0.009 |  |  |  |  |
| N1300 | 0.002 | 0.010 | 0.003 | 0.032 | 0.007 | 0.009 | 0.859 | 0.023 |  |  |  |  |
| N1200 | 0.002 | 0.005 | 0.002 | 0.003 | 0.002 | 0.005 | 0.004 | 0.690 |  |  |  |  |
|  |  |  |  |  |  |  |  |  |  |  |  |  |
|  | D2000 | D1900 | D1800 | D1700 | D1600 | D1400 |  |  |  |  |  |  |
| D2000 | 0.900 | 0.127 | 0.084 | 0.007 | 0.030 | 0.015 |  |  |  |  |  |  |
| D1900 | 0.006 | 0.747 | 0.008 | 0.012 | 0.013 | 0.004 |  |  |  |  |  |  |
| D1800 | 0.042 | 0.079 | 0.864 | 0.015 | 0.008 | 0.004 |  |  |  |  |  |  |
| D1700 | 0.022 | 0.013 | 0.035 | 0.906 | 0.232 | 0.005 |  |  |  |  |  |  |
| D1600 | 0.024 | 0.011 | 0.004 | 0.053 | 0.701 | 0.007 |  |  |  |  |  |  |
| D1400 | 0.005 | 0.023 | 0.005 | 0.007 | 0.016 | 0.965 |  |  |  |  |  |  |
