# Supplementary material for: Landscape Genetic Structure of a Streamside Tree Species Euptelea pleiospermum (Eupteleaceae): Contrasting Roles of River Valley and Mountain Ridge
Source: PLoS One. 2013 Jun 25;8(6):e66928. doi: 10.1371/journal.pone.0066928 (PMC3692547; doi:10.1371/journal.pone.0066928)
Supplement: Table S2 — Historical migration rates between Euptelea pleiospermum populations along each river. (DOC) [file pone.0066928.s002.doc]

**Table S2** Historical migration rates between *Euptelea pleiospermum* populations along each river obtained using the program MIGRATE version 3.2.2.windows (Beerli 2009). (Above diagonal, up-to-down migration rate; below diagonal, down-to-up migration rate).

|  | Y2000 | Y1900 | Y1800 | Y1700 | Y1600 | Y1500 | Y1400 | Y1300 | Y1200 | Y1100 | Y1000 | Y900 |
| --- | --- | --- | --- | --- | --- | --- | --- | --- | --- | --- | --- | --- |
| Y2000 | - | 11.73 | 9.26 | 3.48 | 98.87 | 40.12 | 25.62 | 19.16 | 9.35 | 9.45 | 21.52 | 21.17 |
| Y1900 | 10.47 | - | 11.03 | 16.63 | 11.57 | 37.33 | 21.97 | 16.21 | 10.13 | 16.34 | 16.50 | 5.97 |
| Y1800 | 16.22 | 19.84 | - | 67.19 | 152.37 | 34.25 | 10.83 | 33.10 | 23.87 | 14.38 | 15.44 | 33.53 |
| Y1700 | 13.98 | 12.56 | 10.20 | - | 55.66 | 23.83 | 30.42 | 13.36 | 9.99 | 5.20 | 14.65 | 35.73 |
| Y1600 | 17.09 | 20.07 | 6.94 | 19.37 | - | 117.52 | 34.02 | 7.05 | 4.70 | 6.27 | 22.07 | 18.83 |
| Y1500 | 11.45 | 5.96 | 3.91 | 8.96 | 15.13 | - | 16.13 | 6.42 | 3.77 | 3.60 | 16.31 | 12.42 |
| Y1400 | 17.15 | 7.35 | 3.70 | 13.06 | 24.22 | 32.39 | - | 11.02 | 3.14 | 10.30 | 16.31 | 9.28 |
| Y1300 | 22.74 | 16.05 | 12.89 | 12.65 | 1.50 | 36.32 | 60.06 | - | 11.66 | 6.44 | 26.65 | 18.37 |
| Y1200 | 40.21 | 7.90 | 6.48 | 9.12 | 1.71 | 19.01 | 15.66 | 22.64 | - | 20.79 | 30.92 | 22.75 |
| Y1100 | 18.82 | 37.14 | 15.54 | 19.24 | 23.40 | 35.65 | 13.66 | 67.89 | 25.20 | - | 25.95 | 3.04 |
| Y1000 | 15.71 | 9.65 | 5.16 | 9.98 | 20.31 | 21.79 | 24.52 | 18.73 | 11.07 | 6.22 | - | 94.61 |
| Y900 | 12.74 | 16.54 | 11.50 | 40.17 | 16.31 | 29.50 | 17.70 | 23.06 | 9.13 | 5.22 | 10.43 | - |
|  |  |  |  |  |  |  |  |  |  |  |  |  |
|  | X2000 | X1900 | X1800 | X1700 | X1600 | X1500 | X1400 | X1300 | X1200 | X1100 |  |  |
| X2000 | - | 10.60 | 16.00 | 7.33 | 8.58 | 37.61 | 7.88 | 20.59 | 19.28 | 13.18 |  |  |
| X1900 | 75.36 | - | 21.61 | 24.09 | 20.37 | 32.34 | 17.37 | 31.60 | 6.20 | 10.25 |  |  |
| X1800 | 69.10 | 18.50 | - | 6.28 | 16.76 | 19.03 | 17.65 | 34.20 | 7.20 | 11.04 |  |  |
| X1700 | 31.49 | 19.10 | 20.62 | - | 100.10 | 49.63 | 14.98 | 24.01 | 14.81 | 26.42 |  |  |
| X1600 | 9.54 | 7.22 | 10.47 | 14.07 | - | 25.20 | 8.89 | 13.98 | 25.44 | 17.67 |  |  |
| X1500 | 25.17 | 38.08 | 21.43 | 11.16 | 8.99 | - | 17.14 | 28.50 | 0.84 | 14.56 |  |  |
| X1400 | 10.25 | 20.01 | 46.31 | 15.34 | 44.77 | 3.39 | - | 63.53 | 7.75 | 45.42 |  |  |
| X1300 | 19.43 | 11.41 | 5.90 | 14.20 | 10.82 | 15.88 | 5.60 | - | 20.34 | 14.97 |  |  |
| X1200 | 2.45 | 18.99 | 10.69 | 71.90 | 12.91 | 9.49 | 6.92 | 13.05 | - | 9.40 |  |  |
| X1100 | 42.19 | 34.43 | 9.05 | 26.77 | 17.70 | 13.90 | 13.77 | 19.29 | 13.88 | - |  |  |
|  |  |  |  |  |  |  |  |  |  |  |  |  |
|  | N1900 | N1800 | N1700 | N1600 | N1500 | N1400 | N1300 | N1200 |  |  |  |  |
| N1900 | - | 23.50 | 29.29 | 25.98 | 19.24 | 14.95 | 29.01 | 37.70 |  |  |  |  |
| N1800 | 18.18 | - | 12.80 | 24.40 | 11.03 | 17.83 | 18.40 | 24.58 |  |  |  |  |
| N1700 | 11.12 | 11.97 | - | 10.43 | 10.71 | 27.20 | 28.25 | 16.60 |  |  |  |  |
| N1600 | 11.68 | 4.66 | 28.61 | - | 24.06 | 9.65 | 19.64 | 9.82 |  |  |  |  |
| N1500 | 12.85 | 24.21 | 15.62 | 26.99 | - | 66.71 | 21.53 | 20.09 |  |  |  |  |
| N1400 | 27.51 | 25.37 | 18.24 | 30.87 | 16.43 | - | 40.94 | 20.30 |  |  |  |  |
| N1300 | 27.59 | 19.64 | 35.66 | 6.35 | 17.50 | 11.74 | - | 23.64 |  |  |  |  |
| N1200 | 12.12 | 37.77 | 14.30 | 2.67 | 5.65 | 5.71 | 26.80 | - |  |  |  |  |
|  |  |  |  |  |  |  |  |  |  |  |  |  |
|  | D2000 | D1900 | D1800 | D1700 | D1600 | D1400 |  |  |  |  |  |  |
| D2000 | - | 21.82 | 22.02 | 21.51 | 20.24 | 22.24 |  |  |  |  |  |  |
| D1900 | 18.37 | - | 17.09 | 19.31 | 83.71 | 12.97 |  |  |  |  |  |  |
| D1800 | 43.09 | 32.95 | - | 17.47 | 9.71 | 30.69 |  |  |  |  |  |  |
| D1700 | 22.89 | 17.33 | 21.64 | - | 49.94 | 20.11 |  |  |  |  |  |  |
| D1600 | 15.30 | 22.63 | 11.61 | 5.69 | - | 13.97 |  |  |  |  |  |  |
| D1400 | 77.60 | 26.89 | 18.65 | 14.28 | 16.43 | - |  |  |  |  |  |  |
